# Application of a Novel Alkali-Tolerant Thermostable DyP-Type Peroxidase from *Saccharomonospora viridis* DSM 43017 in Biobleaching of Eucalyptus Kraft Pulp

**DOI:** 10.1371/journal.pone.0110319

**Published:** 2014-10-21

**Authors:** Wangning Yu, Weina Liu, Huoqing Huang, Fei Zheng, Xiaoyu Wang, Yuying Wu, Kangjia Li, Xiangming Xie, Yi Jin

**Affiliations:** 1 College of Biological Sciences and Technology, Beijing Forestry University, Beijing, PR China; 2 Feed Research Institute Chinese Academy of Agricultural Sciences, Beijing, PR China; 3 College of Materials Science and Technology, Beijing Forestry University, Beijing, PR China; University of Milan, Italy

## Abstract

*Saccharomonospora viridis* is a thermophilic actinomycete that may have biotechnological applications because of its dye decolorizing activity, though the enzymatic oxidative system responsible for this activity remains elusive. Bioinformatic analysis revealed a DyP-type peroxidase gene in the genome of *S. viridis* DSM 43017 with sequence similarity to peroxidase from dye-decolorizing microbes. This gene, *svidyp*, consists of 1,215 bp encoding a polypeptide of 404 amino acids. The gene encoding *Svi*DyP was cloned, heterologously expressed in *Escherichia coli*, and then purified. The recombinant protein could efficiently decolorize several triarylmethane dyes, anthraquinonic and azo dyes under neutral to alkaline conditions. The optimum pH and temperature for *Svi*DyP was pH 7.0 and 70°C, respectively. Compared with other DyP-type peroxidases, *Svi*DyP was more active at high temperatures, retaining>63% of its maximum activity at 50–80°C. It also showed broad pH adaptability (>35% activity at pH 4.0–9.0) and alkali-tolerance (>80% activity after incubation at pH 5–10 for 1 h at 37°C), and was highly thermostable (>60% activity after incubation at 70°C for 2 h at pH 7.0). *Svi*DyP had an accelerated action during the biobleaching of eucalyptus kraft pulp, resulting in a 21.8% reduction in kappa number and an increase of 2.98% (ISO) in brightness. These favorable properties make *Svi*DyP peroxidase a promising enzyme for use in the pulp and paper industries.

## Introduction

Heme peroxidases, containing prosthetic heme groups, have been divided into two large groups, animal and plant peroxidases [1]. The plant peroxidases are divided into classes I, II, and III, which include fungal (class II) and bacterial peroxidases (class I), according to primary structural homology [2]. Class I peroxidases contain mitochondrial yeast cytochrome c peroxidase, chloroplast and cytosol ascorbate peroxidases, and gene duplicated bacterial peroxidase [2]. Class II enzymes include secretory fungal peroxidases, such as lignin peroxidase [3], manganese peroxidase [4], and versatile peroxidase [5]. Secretory plant peroxidases such as horseradish peroxidase and barley grain peroxidase belong to class III peroxidases. The recently described DyP-type peroxidases (DyPs) (EC 1.11.1.19) superfamily comprises a novel class of heme peroxidases, and are found in both fungi and bacteria [6]–[14]. DyPs have been classified into four phylogenetically distinct subfamilies, with fungal enzymes belonging to subfamily D and bacterial enzymes constituting subfamilies A–C [15], [16]. These enzymes are very promising for use in biotechnological applications. Despite the large number of putative DyP sequences registered in PeroxiBase (http://peroxibase.toulouse.inra.fr/), only a few studies have described the isolation and characterization of peroxidases, including four proteins from actinomycetes [17]-[21]. However, no DyP-type peroxidase genes have been identified in *Pseudonocardiaceae* to date.

Biobleaching is the bleaching of pulps using ligninolytic microorganisms or enzymes that reduce the amount of chemical bleach required to obtain a desirable brightness of pulps. Some enzymes from fungi and bacteria, such as extracellular xylanase and peroxidase from *Streptomyces* sp [22], [23], laccase and manganese peroxidase produced by *Trametes versicolor* [24], [25], have been studied for biobleaching of paper pulp and other industrial applications, as they degraded lignin or xylan and decolorized the pulp [26], [27]. However, no DyP-type peroxidase enzymes has been characterized and applied in the pulp bleaching field.

In the current work, a novel peroxidase, *Svi*DyP, related to a putative DyP-type peroxidase enzyme, was isolated, purified, and characterized from *Saccharomonospora viridis*, a pentachlorophenol-degrading thermophilic actinomycete [28]. In addition, the catalytic properties of the enzyme and its application as an enzymatic pretreatment for kraft pulp bleaching were investigated. *Svi*DyP was an alkali-tolerant thermostable dye-decolorizing peroxidase reported from the *Pseudonocardiaceae* family, which belongs to the genus of *Saccharomonospora*, and it had an accelerated action during the biobleaching of eucalyptus kraft pulp. These favorable properties made *Svi*DyP a good candidate for application in the pulp and paper industries.

## Materials and Methods

### Microorganism, culture conditions, and chemicals

*Saccharomonospora viridis* DSM 43017 was obtained from the China General Microbiological Culture Collection Center, Beijing (reference number CGMCC 4.1324). The strain was cultivated in a shaker flask at 45°C in STS medium (1.0% (w/v) soy peptone, 1.0% (w/v) glucose, 0.2% (w/v) yeast extract, 0.2% (w/v) NaCl, and 0.2% casein enzymatic hydrolysate, all from Biodee, Beijing, China), adjusted pH to 8.0 with NaOH prior to autoclaving.

*Escherichia coli* DH5α and *E. coli* BL21 (Tiangen, Beijing, China) (were cultivated in Luria Bertani (LB) medium at 37°C for gene cloning, sequencing, and expression. The pEASY-T3 vector (TransGen, Beijing, China) was used for plasmid gene cloning and sequencing. The plasmid pET-28a (+) (Takara Bio, Otsu, Japan) was used as an expression vector. Manganese peroxidase (MnP), azure B, brilliant green, reactive blue 19, reactive green 19, reactive yellow 2, reactive black 5, reactive red 120, malachite green and crystal violet were purchased from Sigma (St. Louis, MO, USA). All other reagents used were of analytical grade unless otherwise stated.

### Decolorization assay for *S. viridis* on solid medium

Azure B, which was membrane-filtered with a 0.45-µm cellulose nitrate filter, was mixed with STS medium to give a final concentration of 0.1 g l^−1^ and used to make agar plates. *S. viridis* was inoculated onto the plates with a sterile spreader. The plates were then incubated at 45°C and their surface appearance was observed daily. A ring of decoloration around the colonies on the blue medium is a qualitative signal of presence or absence of dye-decolorizing enzyme DyP-type peroxidase. The presence of bleaching around colonies on the blue medium indicates the production of the dye-decolorizing enzyme.

### Bioinformatic and phylogenetic analysis and homology modeling

The whole genome of *S. viridis* DSM 43017 is available from the GenBank database under the accession number NC_013159 [29]. By performing a sequence homology comparison between four well characterized DyP-type peroxidases from *Shewanella oneidensis* TyrA [13], *Bacteroides thetaiotaomicron* BtDyP [30], *Rhodococcus jostii* DyPB [31], and *E. coli* YcdB [32], one unannotated DyP-type peroxidase gene (Gene YP_003135694) was identified in the genome of *S. viridis* DSM 43017. The open reading frame, which we tentatively named *svidyp*, showed sequence similarity to peroxidase genes in other dye-decolorizing microbes. The DNA sequence of *svidyp* was analyzed using the software package DNAMAN 6.0 (Lynnon Corporation, Quebec, Canada). Nucleotide and the corresponding deduced amino acid sequence homology searches were carried out using the BLASTn and BLASTp programs, respectively, at the NCBI website (http://www.ncbi.nlm.nih.gov/BLAST/). Multiple sequence alignment was performed by ClustalW [33]. A phylogenetic tree containing the closest homologs of the DyP protein was constructed using MEGA 5.2 with the neighbor-joining algorithm [34]. The deduced tertiary structure of *Svi*DyP was derived from the amino acid sequence using SWISS-MODEL [35]–[37].

### Gene cloning, heterologous expression and enzyme purification

Genomic DNA from *S. viridis* was isolated following overnight growth in STS liquid medium. Cells were harvested by centrifugation (10,000×*g*, 25°C for 2 min). DNA extraction was then performed using the Bacterial Genomic DNA Extraction Kit V.3.0 (Takara) according to the manufacturer's instructions. Two primers were designed at the 5′ and 3′ ends of the *svidyp* gene with the following sequences: Svidyp-F: 5′-GGCGAATTCATGAAGGGCCGGCGGTTC-3′ and Svidyp-R: 5′-GCCAAGCTTTCAGGCTTCCAACAGCGG-3′, which contained the restriction sites *Eco*RI and *Hin*dIII (underlined), respectively, to enable directional cloning into the pET-28a (+) expression vector. Using these primers, the complete *svidyp* gene was PCR-amplified from *S*. *viridis* genomic DNA. PCR amplification was performed using *Taq* DNA polymerase, 10× PCR buffer (Mg^2+^ Plus), and dNTP mixture (Takara) under the following conditions: 5 cycles of 94°C for 30 s, 67.3°C for 30 s, and 72°C for 1 min, followed by 30 cycles of 94°C for 30 s, 63.7°C for 30 s, and 72°C for 1 min.

The resulting PCR product was gel-purified, digested with *Eco*RI and *Hin*dIII, and cloned into the corresponding site of the pET28a (+) vector to construct the recombinant plasmid pET28a-svidyp. The plasmid was then transformed into chemically competent *E. coli* BL21 cells. Plasmid DNA from recombinant *E. coli* was isolated using a Plasmid Purification Kit (Takara). DNA concentration was estimated by the absorbance at 260 nm, and DNA integrity was verified by agarose gel electrophoresis. Transformants were identified by PCR analysis and enzymatic digestion, both as described above, and were then confirmed by DNA sequencing using the primers T7 and T7-terminor. Sequencing was carried out by Beijing AuGCT DNA-SYN Biotechnology Co., Beijing, China.

A positive transformant harboring pET28a-svidyp was isolated as a single colony for gene expression. The transformant was cultured overnight at 37°C in 10 ml LB medium containing kanamycin at 50 µg ml^−1^. The culture was then inoculated into fresh LB medium (1∶100 dilutions) containing kanamycin and was grown at 37°C to an OD_600_ of approximately 0.6. Isopropyl-β-D-1-thiogalactopyranoside (IPTG) was then added to a final concentration of 1.0 mM for induction, and the culture was further cultivated at different temperature and time. Following induction with 1.0 mM IPTG at 25°C for 3 h, little activity was detected from the cell lysate, while incubation at 16°C for 12 h the activity in the lysate increased to 9.4 U ml^−1^. Thus, to effectively induce enzyme expression, induction with 1.0 mM IPTG at 16°C overnight was selected for further production of the recombinant DyP peroxidise. No DyP peroxidase activity was detected from the uninduced transformant or from the transformant harboring the empty pET-28a (+) vector.

Cultures of induced recombinant *E. coli* (200 ml) were centrifuged (10,000×*g*, 4°C for 10 min) and the cell pellets were resuspended in 10 ml sodium phosphate buffer (50 mM, pH 8.0). The cell suspension was then lysed by sonication (pause on: 10 s; pause off: 10 s; power: 130 W; time: 30 min) using a cell ultra sonicator (Sonics* VCX130; Sonics & Materials, CT, USA). Following sonication, the unbroken cells and large debris were removed by centrifugation (12,000×*g*, 4°C for 20 min) and the supernatant was collected. Because the enzyme was expressed as a His-tagged protein, the supernatant was loaded onto a Ni-affinity column (Ni–NTA Agarose; Qiagen, Valencia, CA, USA) and eluted with a linear gradient of imidazole (20–250 mM, 25 ml) in sodium phosphate buffer (20 mM phosphate, 300 mM NaCl, pH 8.0), at a flow rate of 1 ml min^−1^. Fractions displaying DyP-peroxidase activity were combined and applied to a Sephadex-G75 (10×200 mm) column pre-equilibrated with 0.1 M phosphate buffer (pH 7.0) and eluted with the same buffer at a flow rate of 0.5 ml min^−1^. The purified enzyme with purification yields of 40.3% (Table 1) was stored at −20°C until further characterization. The protein concentration was determined using a Bradford assay [38] using bovine serum albumin as a standard. The purity of *Svi*DyP was verified by sodium dodecyl sulfate polyacrylamide gel electrophoresis (SDS-PAGE) analysis on a 12% gel as described previously [39] and by Western blotting analysis using an anti-His6 tag mouse monoclonal antibody [40].

**Table 1:** Purification of recombinant *Svi*DyP.

Step	Activity (U)	Protein (mg)	Specific activity (U mg^−1^)	Yield (%)	Purification fold
Lysate supernatant	940.0	1175	0.8	100.0	1.0
Ni-NTA agarose	437.9	39.1	11.2	46.6	14.0
Sephadex G-75	379.1	21.3	17.8	40.3	22.3

### Enzyme assays

Brilliant green, a representative triarylmethane dye, was used as the substrate. The substrate solution consisted of 125 µM brilliant green in 50 mM phosphate buffer (pH 7.0). One hundred microliters of the purified *Svi*DyP enzyme solution (0.01 mg ml^−1^) was mixed with the substrate solution. All reactions were started by addition of H_2_O_2_ (0.1 mM for all tests). The total volume of the enzyme reaction mixture was then adjusted to 3 ml. The enzyme activity was calculated by measuring the variation in absorbance at 623 nm using the molar extinction coefficient of brilliant green (max. wavelength: 623 nm; ε_623_ = 18,000M^−1^cm^−1^). One unit of enzyme activity was defined as the amount of enzyme that decolorized 1 µmol of brilliant green at 60°C in 1 min [41]. The same method was used to study the substrate range of the enzyme using reactive blue 19 (max. wavelength: 590 nm; ε_590_ = 10,000 M^−1^ cm^−1^), reactive green 19 (max. wavelength: 630 nm; ε_630_ = 22,000 M^−1^ cm^−1^), reactive yellow 2 (max. wavelength: 390 nm; ε_390_ = 8,000 M^−1^ cm^−1^), reactive black 5 (max. wavelength: 598 nm; ε_598_ = 30,000 M^−1^ cm^−1^), reactive red 120 (max. wavelength: 535 nm; ε_535_ = 28,000 M^−1^ cm^−1^), malachite green (max. wavelength: 617 nm; ε_617_ = 8,400 M^−1^ cm^−1^), crystal violet (max. wavelength: 583 nm; ε_583_ = 21,900 M^−1^ cm^−1^), azure B (max. wavelength: 651 nm; ε_630_ = 22,000 M^−1^ cm^−1^), 2, 6 - Dimethoxyphenol (max. wavelength: 469 nm; ε_469_ = 2,750 M^−1^ cm^−1^) and veratryl alcohol (max. wavelength: 310 nm; ε_310_ = 9,300 M^−1^ cm^−1^) as substrates under their corresponding maximum absorption wavelength. Control treatments without H_2_O_2_ and/or without enzyme were performed. MnP activity was measured at 25°C by the formation of Mn^+3^-tartrate complex (ε_238_ = 6,500 M^−1^ cm^−1^) during the oxidation of 0.5 mM MnSO_4_ in 100 mM tartrate bufferat pH 4.5 with 0.1 mM H_2_O_2_ [8]. One unit of MnP activity corresponds to the amount of enzyme which oxidizes 1 µmol Mn^2+^ to Mn^3+^ per minute at pH 4.5 and 25°C (in the presence of 0.1 mM H_2_O_2_).

### Enzyme characterization

Aliquots of the recombinant *Svi*DyP sample (1 mg ml^−1^) were put under various conditions to test its stability. The optimal pH for enzyme activity was determined by measuring the reaction rate in buffers covering a pH range of 3.0–12.0. The buffers used were 50 mM citric acid-Na_2_HPO_4_ (pH 3.0–8.0), 50 mM Tris-HCl (pH 8.0–9.0), and 50 mM glycine-NaOH (pH 9.0–12.0). The pH stability was measured by adding enzyme to buffers ranging from pH 2.0–10.0 at 37°C for 1 h without the substrate solutions, and then, its residual activity was analyzed as described above. The highest activity of the enzyme, treated as above, was defined as 100%. The optimal temperature for enzyme activity was examined over the temperature range of 30–90°C. To estimate the thermal stability, the enzymes were incubated at 50°C, 60°C, 70°C, or 80°C for 0.5, 1, 2, 3, 4, and 5 h without substrate, and the residual enzyme activity was measured with the method as described above. To determine the effect of different metal ions and chemical reagents on the enzyme activity, the enzyme sample (1 mg ml^−1^) was incubated for 30 min at 37°C and pH 7.0 in 0.05 M Britton–Robinson buffer, supplemented with the following additives respectively (final concentration, 5 mM): SDS, β-mercaptoethanol, ethylenediaminetetraacetic acid (EDTA), ZnCl_2_, BaCl_2_, MnCl_2_, MgCl_2_, AlCl_3_, CoCl_2_, CaCl_2_, LiCl, FeCl_2_, and FeCl_3_. The residual activity was then assayed using the method as described above. And a system without any additives was used as a control.

The *K*_m_, and *V*_max_ values for the purified *Svi*DyP protein were determined by several substrates including reactive blue 19, reactive yellow 2 and brilliant green. Lineweaver-Burk plots were generated from the initial rates obtained at varying substrate concentrations with a constant concentration of the second substrate [42]. Each experiment was repeated three times and each experiment included three replicates.

### Physico-chemical characterization of kraft pulps and biobleaching with *Svi*DyP

An unbleached eucalyptus kraft pulp was prepared by cooking the dried wood material (provided by JinHai Pulp & Paper Limited Company, Hainan, China) with sulfate process under the following conditions: total alkali, 16.0% (w/v), sulfureted degree 25% (w/v), liquid ratio 1∶6, and a cooking temperature of 160°C for 2 h. The optimization of enzyme dose and reaction time for biobleaching was carried out by treating pulp with varying doses of DyP-type peroxidase, ranging from 0–6.0 U g^−1^ dry pulp, for various time intervals up to 6 h. Pulp properties were investigated at regular intervals. Enzyme-treated and untreated pulp samples at 10% pulp consistency were incubated at 65°C with *Svi*DyP (5 milligram protein per gram dried pulp, equivalently enzyme dosage as 3 U g^−1^ which was in terms of enzyme activity towards brilliant green) in 50 mM citric acid-Na_2_HPO_4_ buffer (pH 7.0) for 2 h, whereas the biobleaching by MnP with 0.5 mM MnSO_4_ was performed at 25°C in pH 4.5 for 2 h as the positive control. 0.1 mM H_2_O_2_ was added in all the assays. During the bleaching treatment, kneading the pulp mixture every 15 min was needed in order to make sure all ingredients can mix well. After the enzyme bleaching, pulps were thoroughly washed with deionized water to remove soluble reducing sugars and any free soluble residual lignin, and then made into pieces of paper by the sheet forming machine. Pulp sheet preparation was conducted as described in the paper [43]. Degree of brightness of pulp according to ISO (International Standards Organization) was measured on an YQ-Z-48A (Qingtong, Hangzhou, China) brightness color tester. Pulp kappa number was determined by using TAPPI useful method T236 (Technical Association of the Pulp and Paper Industry, Atlanta, Ga.). All reported data are averages of experiments performed in at least six replicates.

### Scanning electron microscopy

Samples of pulp fibers were processed for scanning electron microscopy (SEM). Paper pulp samples were formed into hand sheets using a Buchner funnel. Small pieces of fiber were vacuum dried in the presence of P_2_O_5_, placed on the stubs and mounted with silver tape, and then sputter coated with gold using fine coat. The samples were examined at 5.0 kV under a scanning electron microscope (Hitachi S-4300, Ibaraki, Japan) at various magnifications.

## Results

### Decolorization assay for *S. viridis* on solid medium

**Figure 1:**
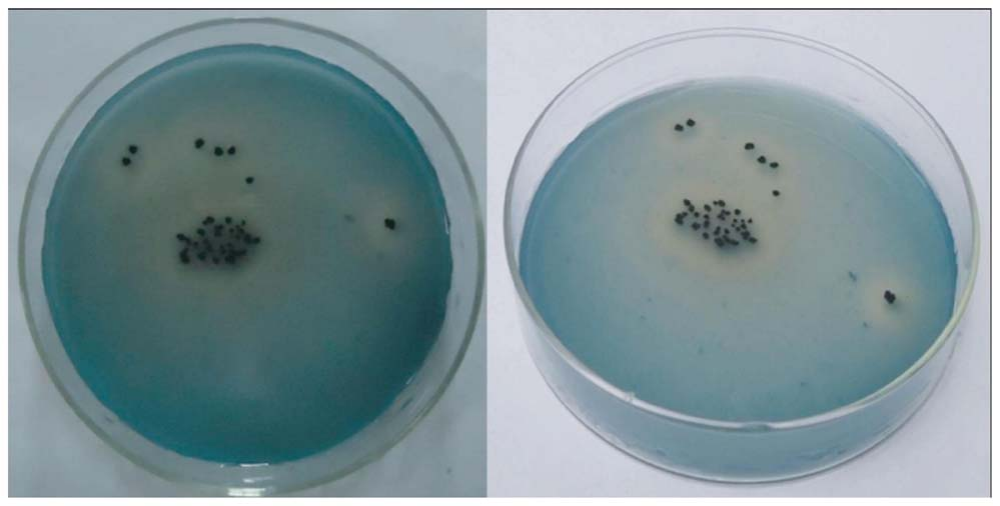
Degradation circles produced by *Saccharomonospora viridis* on azure B screening plates.

Some bleaching laps were observed on STS medium mixed with 0.1 g l^−1^ azure B around colonies of *S. viridis* (Fig. 1), which were produced after incubating at 45°C for 3 days, indicating the presence of a dye-decolorizing enzyme. This indicated one or more extracellular dye-decolorizing enzymes produced in the cultivating process of *S. viridis*.

### *Svi*DyP is a member of the DyP-type family enzymes

To isolate the gene encoding the DyP-type peroxidase from *S. viridis* DSM 43017, two specific primers, Svidyp-F and Svidyp-R, were designed. PCR amplification of the DyP-peroxidase gene (GenBank under accession number KF444221) of *S. viridis* generated a DNA fragment of 1215 bp, which encoded a polypeptide of 404 amino acids with a predicted molecular mass of 44.5 kDa and a deduced isoelectric point of 5.17. Sequence analysis showed that overall G + C content of the *svidyp* gene was 70.1%.

**Figure 2:**
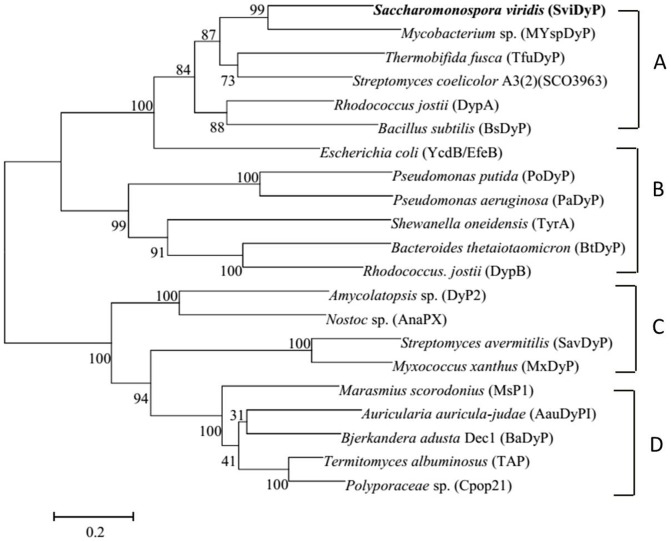
Phylogenetic tree analysis. Phylogenetic tree showing the relationship between *Svi*DyP and other isolated DyPs belonging to subfamilies A–D: MYspDyP from *Mycobacterium* sp. (GI: 126434170), *Tfu*DyP from *Thermobifida fusca* (GI: 71917209), Sco3963 from *Streptomyces coelicolor* A3(2) (GI: 541881521), DypA from *Rhodococcus jostii* (GI: 499917154), *Bs*DyP from *Bacillus subtilis* (GI: 732344), EfeB/YcdB from *Escherichia coli* (GI: 2506638), *Po*DyP from *Pseudomonas putida* (GI: 501229266), *Pa*DyP from *Pseudomonas aeruginosa* (GI: 94829180), TyrA from *Shewanella oneidensis* (GI: 119390160), *Bt*DyP from *Bacteroides thetaiotaomicron* (GI: 109158102), DypB from *R. jostii* (GI: 330689635), DyP2 from *Amycolatopsis* sp. (GI: 409973917), AnaPX from *Nostoc* sp. (GI: 75704119), *Sav*DyP from *Streptomyces avermitilis* (GI: 29604188), *Mx*DyP from *Myxococcus xanthus* (GI: 108465542), Msp1 from *Marasmius scorodonius* (GI: 261266601), *Aau*DyPI from *Auricularia auricula-judae* (GI: 433286646), TAP from *Termitomyces albuminosus* (GI: 20386144), and Cpop21 from *Polyporaceae* sp. (GI: 2160705). Confidence was evaluated with 1000 bootstrap replicates (values are indicated at branch points).

**Figure 3:**
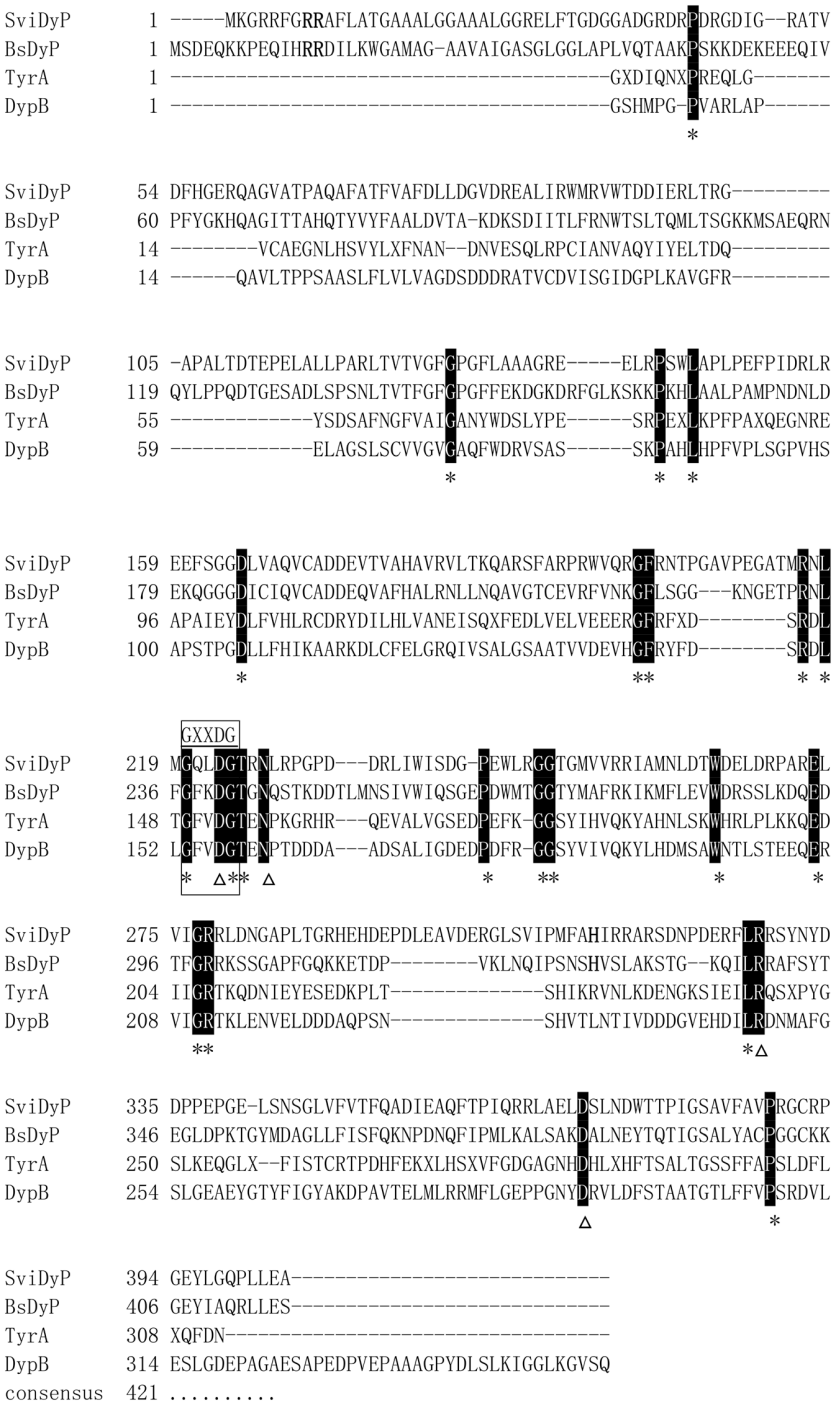
Multiple sequence alignment of the *Svi*DyP amino acid sequence with other members of DyP-type peroxidases family. The sequence alignments were performed using ClustalW (Thompson et al. 1994). Perfect match residues are displayed in white on black shade. The unique GXXDG motif is boxed. The conserved residues of the active site are indicated by asterisks. Residues indicated by triangles are residuals important for coordinating the heme at the active site.

The deduced amino acid sequence of *Svi*DyP showed 36% amino acids identity with *Bacillus subtilis* BsDyP [15] and *R. jostii* DyPB [31], 33% *E. coli* YcdB [32], 27% to *S. oneidensis* TyrA [13] and 21% to *B. thetaiotaomicron* BtDyP [30]. Twenty typical and hotspot DyPs proteins, such as *Thermobifida fusca* TfuDyP [21], *S. oneidensis* TyrA [13], *B. subtilis* BsDyP [15], *and R. jostii* DyPB [31], were used to phylogenetic analysis. Phylogenetic trees and multiple sequence alignments showed that *Svi*DyP was most closely related to *Mycobacterium* sp. *My*spDyP, *Thermobifida fusca Tfu*DyP, *Rhodococcus jostii* DyPA, *Escherichia coli* EfeB/YcdB, *Bacillus subtilis Bs*DyP [15] and the putative uncharacterized protein Sco3963 from *Streptomyces coelicolor* A3 (2), all of which belong to subfamily A (Fig 2). Therefore the enzyme *Svi*DyP was grouped into subfamily A, and typical motifs, such as conserved GXXDG motif and some conserved amino acid residues (D223, N227, R328, H313, E342, D371), were found in DyP-type peroxidases (Fig 3). The model structure of *Svi*DyP showed two domains consisting of a four-stranded antiparallel β-sheet and peripheral α-helices which is typical in the DyP-type family (Figure S1).

### Heterologous expression and purification of *Svi*DyP

**Figure 4:**
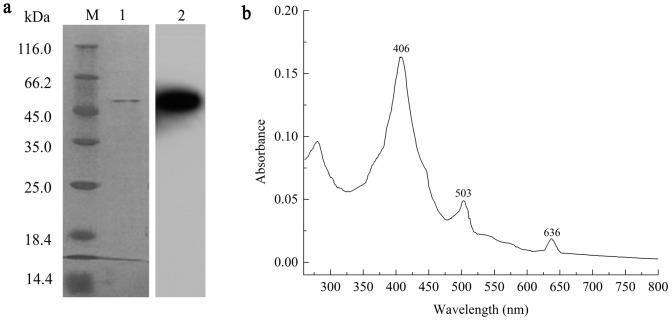
SDS–PAGE and western blotting analysis and spectral characteristics of purified *Svi*DyP. **a**. SDS–PAGE and western blotting analysis. Lanes: M, molecular mass standard; (1) SDS–PAGE analysis of purified *Svi*DyP. (2) western blot analysis of the purified *Svi*DyP recognized by an anti-His6 tag mouse monoclonal antibody. **b**. Spectral characteristics of purified *Svi*DyP, as determined by hemoprotein assay. Purified *Svi*DyP (1 mg ml^−1^) in 25 mM phosphate buffer (pH 7.0) was scanned at 300–800 nm to identify the Soret band.

The svidyp gene was successfully cloned into the pET28a vector and expressed in *E. coli* BL21, forming the recombinant clone *E. coli* BL21/pET28a-svidyp, with the expression of His-tagged protein. The purified *Svi*DyP protein, as verified by SDS-PAGE analysis and Western Blot analysis (Fig. 4a) by anti-6xHis antibody, was about 45 kDa, consistent with the theoretical value of 44.5 kDa.

### Biochemical and spectroscopic characterization of *Svi*DyP

The UV-visible spectrum of *Svi*DyP at resting state showed the main Soret band at 406 nm and two charge transfer bands at 503 nm and 636 nm (Fig. 4b), as well as a Reinheitszahl ratio (A_406_/A_280_) of 1.7. The molar absorption coefficient of *Svi*DyP, ε_406,_ was 97,000 M^−1^ cm^−1^, similar to values found for other peroxidases and DyPs [8], [44].

The recombinant enzyme expressed and purified from *E. coli* BL21 was then biochemically characterized. The controls of without H_2_O_2_ and/or without enzyme were without significant statistical difference. Substrate specificity of the enzyme was determined by using a subset of well-known peroxidase substrates and anthraquinone and azo dyes (Table 2). The enzyme displayed maximal activity towards the anthraquinone dye reactive blue 19 (1.29 U mg^−1^), the azo dyes reactive green 19, reactive yellow 2, reactive black 5, and reactive red 120 (1.32, 4.86, 0.96 and 0.69 U mg^−1^, respectively), and the triarylmethane dyes brilliant green, malachite green, and crystal violet (12.24, 8.4 and 4.11 U mg^−1^, respectively). *Svi*DyP was able to act on veratryl alcohol (0.03 U mg^−1^), but not very efficiently (Table 2), and also showed activity towards azure B (1.62 U mg^−1^), a methylated thiazine dye that is a good substrate for lignin peroxidase [45]. Additionally, *Svi*DyP showed a modest activity towards 2, 6 - dimethoxyphenol (0.06 U mg^−1^) which is a typical methoxylated phenol substrate for plant peroxidases. From the above, it is true that *Svi*DyP displays (DyP-type) peroxidase activity.

**Table 2:** Enzymatic activity of purified *Svi*DyP towards various dyes^a^.

Substrate	λ_max_ (nm)/ε at λ_max_ b (M^−1^ cm^−1^)	pH	Temp (°C)	Initial concn (µM)	Specificity activity (U mg^−1^)^c^	Relative activity (%)^d^
Reactive Blue 19^e^	590/10000	7.0	70	50	1.29	10.5
Reactive Green 19^f^	630/22000	7.0	60	35	1.32	10.8
Reactive Yellow 2^f^	390/8000	7.0	60	125	4.86	39.7
Reactive Black 5^f^	598/30000	7.0	60	12.5	0.96	7.8
Reactive Red 120^f^	535/28000	7.0	60	12.5	0.69	5.6
Brilliant Green^g^	623/18000	7.0	70	12.5	12.24	100
Malachite Green^g^	617/8400	7.0	70	32	8.4	68.6
Crystal Violet^g^	583/21900	7.0	70	50	4.11	33.6
Azure B	651/8700	6.0	70	32	1.62	13.2
2,6-Dimethoxyphenol	469/2750	4.5	60	100	0.06	0.5
Veratryl alcohol	310/9300	4.5	60	100	0.03	0.2

a The total volume of the enzyme reaction mixture was 3 ml.

b Molar extinction coefficient at maximum absorption wavelength (λmax) of each dye.

c Activity was calculated as specific activity in U mg^−1^ (1 U = 1 µmol/min).

d Relative activity was defined as activity towards brilliant green.

e AQ, anthraquinone.

f AZ, azo.

g TM, triarylmethane.

**Figure 5:**
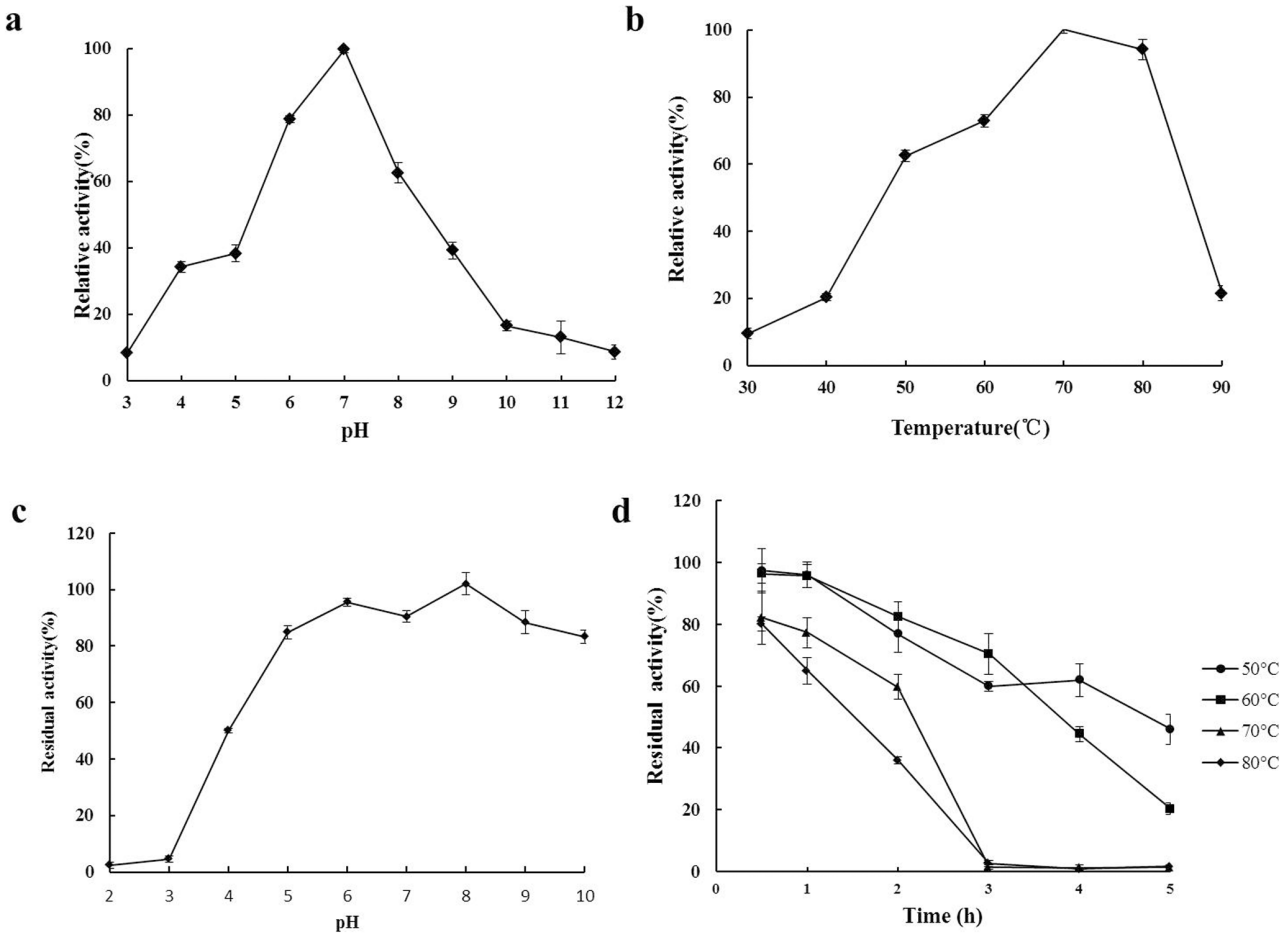
Characterization of *Svi*DyP. **a**. pH optimization. The enzyme was assayed at different pH, as described in the “Materials and Methods”section. **b**. Temperature optimization. The enzyme samples were incubated in pH 7.0 at 30, 40, 50, 60, 70, 80, and 90°C for 30 min and their residual activities were assayed, as described in the “Materials and Methods”section. **c**. pH stability. Enzyme samples (1 mg ml^−1^) were incubated for 30 min at different pH levels at 37°C, and then their residual activities were assayed at the optimum pH,as described in the “Materials and Methods” section. **d**. Temperature stability. The enzyme samples were incubated in pH 7.0 at 50, 60, 70, or 80°C, and then their residual activities were assayed at different times.

To study the behavior of the enzyme under various pH and temperature conditions, brilliant green was used as the substrate. The optimal pH of *Svi*DyP at 60°C was 7.0. It had broad pH adaptability (>35% activity at pH 4.0–9.0) and retained over 62% of the peak activity at pH 6.0–8.0 (Fig. 5a). *Svi*DyP was highly alkali-tolerant, retaining at least 80% of its initial activity after incubation at 37°C for 1 h in buffers ranging from pH 5.0–10.0 (Fig. 5b). The optimal temperature for *Svi*DyP activity was 70°C, at pH 7.0 (Fig. 5c). *Svi*DyP was highly thermophilic, retaining at least 63% of its maximum activity at 50–80°C, and approximately 95% at 80°C. The thermostability of the enzyme was investigated for a period of 5 h at temperatures ranging from 50–80°C (Fig. 5d). It was observed that *Svi*DyP maintained approximately 60% of maximal activity at 60°C and 70°C for at least 3 h and 2 h, respectively.

The effect of different metal ions and metal chelating agents on the enzymatic activity was also assayed, using brilliant green as the substrate (Table 3). The activity of control without any additives was defined as 100%. The activity of the enzyme was not affected (residual activity,>80%) by the presence of 5 mM final concentration of Ba^2+^, Mn^2+^, Mg^2+^, Fe^2+^, or EDTA. An obvious reduction in catalytic activity was found in solutions containing 5 mM Fe^3+^ or SDS. Al^3+^ and Co^2+^ caused 27.6% and 16.9% activation of *Svi*DyP, respectively. The addition of 5 mM Cu^2+^, Zn^2+^, Ca^2+^, or Li^+^ had similar but less activating effects on the activity of *Svi*DyP, probably through promoting an oxidation reaction. β-mercaptoethanol highly boosted the enzymatic activity by almost a factor of 8.5.

**Table 3:** Effect of various agents on *Svi*DyP activity.

Reagent	Residual activity (%) 5 mM
None	100
Zn^2+^	102.3±2.0
Ba^2+^	99.6±1.7
Mn^2+^	92.3±2.1
Mg^2+^	96.4±3.1
Al^3+^	127.6±3.0
Co^2+^	116.9±2.2
Ca^2+^	84.7±4.1
Li^+^	87.0±3.3
Fe^2+^	96.1±4.1
Fe^3+^	35.9±3.9
EDTA	97.2±2.0
β-mercaptoethanol	847.9±1.9
SDS	UD

UD, undetectable activity.

EDTA, ethylenediaminetetraacetic acid.

SDS, sodium dodecyl sulfate.

The kinetic analysis of purified *Svi*DyP corroborated the Michaelis-Menten behavior of the enzyme, with a *V*_max_ of 38.46±0.98 µmol l^−1^ min^−1^ and a *K*_m_ of 58.87±4.7 µmol l^−1^ for brilliant green, a *V*_max_ of 3.7±0.41 µmol l^−1^ min^−1^ and a *K*_m_ of 187.2±16.6 µmol l^−1^ for reactive yellow 2, and a *V*_max_ of 2.2±0.26 µmol l^−1^ min^−1^ and a *K*_m_ of 210.2±16.5 µmol l^−1^ for reactive blue 19. Such kinetic behavior strongly suggested that the cloned enzyme might be a DyP-type peroxidase.

### Enzyme effects on pulp bleaching

Enzyme effects on pulp bleaching were reflected by measuring the physicochemical properties of paper pulp biobleached by enzymic preparations. The unbleached/raw eucalyptus kraft pulp had a kappa number of 11.0 and a brightness of 33.6% (ISO) [43]. With the increase of enzyme dosage and reaction time, the pulp brightness was increased and kappa number was reduced gradually, and then they keep invariant (Figure S2). When the enzyme dosage was 3 U g^−1^ (, b) and the reaction time was 2 h (Figure S2c, d), the pulp brightness reached the maximum, and pulp kappa number reached the lowest value. Therefore, the choice of 3 U g^−1^ as an optimal enzyme dosage and 2 h as an optimal reaction time were the best condition of *Svi*DyP biobleaching kraft pulp (Figure S2). The experiments of pulp bleaching by recombinant protein *Svi*DyP showed that enzyme treatment to improve the brightness of the pulp 2.98%, pulp kappa number reduced 21.8% compared with control (Table 4), indicating that the enzyme treatment can significantly improve the brightness of the pulp, but reduce kappa value will help subsequent bleaching. Application of MnP in the pulp bleaching experiment showed that MnP reduced the specific pulp kappa number by 31.8%, and increased the whiteness of pulp by 5.90% at pH 4.5, 25°C (Table 4), whereas it reduced the specific pulp kappa number by 24.8% and increased the whiteness of pulp by 3.26% at pH 7, 65°C.

**Table 4:** Physicochemical properties of enzyme-treated eucalyptus kraft pulp.

Enzyme (Treatment)	Samples	Brightness (% ISO)	Brightness increase (%)	Kappa number	Kappa decrease (%)
*Svi*DyP	Control^a^	33.6±0.1	-	11.0±0.2	-
(65°C pH 7.0)	Test	34.6±0.4	2.98	8.6±0.1	21.8
MnP	Control	33.7±0.4	-	10.9±0.3	-
(65°C pH 7.0)	Test	34.8±0.3	3.26	8.2±0.1	24.8
MnP	Control	33.9±0.2	-	11.0±0.4	-
(25°C pH 4.5)	Test	35.9±0.2	5.90	7.5±0.2	31.8

a The control samples and the test samples were incubated in the selected buffer at the set temperature with and without corresponding enzyme.

**Figure 6:**
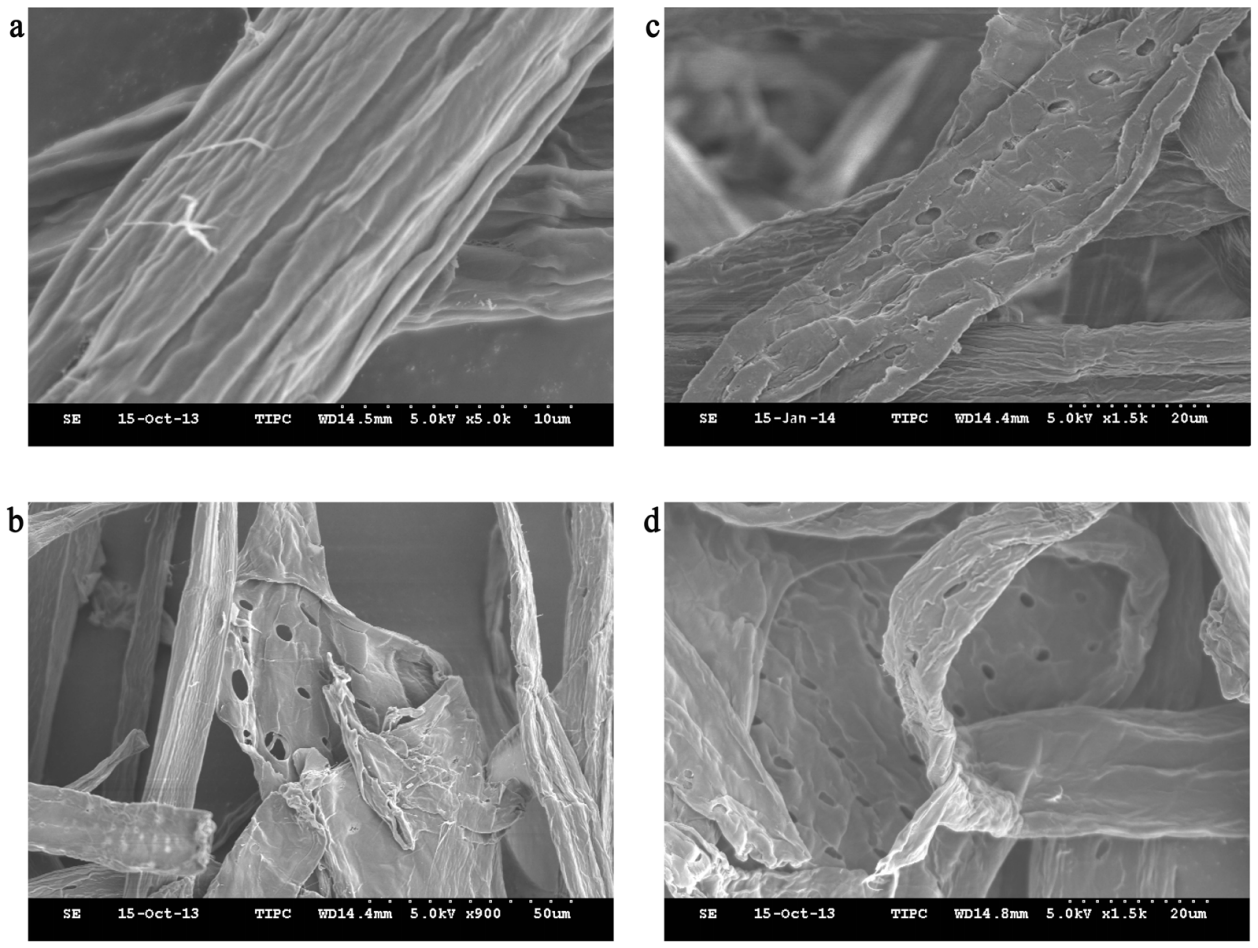
Morphology of bleached pulp fiber surface. Scanning electron micrograph of unbleached (control) eucalyptus kraft pulp (**a**). Scanning electron micrograph of MnP treated eucalyptus kraft pulp (**b**). Scanning electron micrograph of *Svi*DyP treated eucalyptus kraft pulp (**c**, **d**).

To study the effect of the enzyme on fiber surface morphology in *Svi*DyP-bleached pulp, eucalyptus kraft pulp fiber morphology and surface changes were observed by SEM (Fig. 6). Without any enzymatic bleaching, eucalyptus kraft pulp fibers were stiff, and the surface structure was relatively smooth with no damage or cracks (Fig. 6a). The surface morphology of fibers treated with MnP is shown in Fig. 6b and the fibers treated with DyP-type peroxidase from *S. viridis* are shown in Fig. 6c and 6d. The pulp fiber surface treated with DyP-type peroxidase from *S. viridis* appeared rough and wrinkled and showed signs of slight fissures as well as that treated with MnP. There were also many holes in the surface fibers, and fibers began to increase in flexibility (Fig. 6b, 6c and 6d). These representative images show the obvious cracks, grooves and spalling in the surface of the fibers, with some fibers having holes with an aperture diameter of 7 µm, which increases fiber flexibility.

## Discussion

The pentachlorophenol-degrading thermophilic actinomycete *S. viridis* and its extracellular enzymes offer great potential for different biotechnological applications. Both a crude extracellular xylanase [46] and a purified xylanase [47], apparently in the absence of cellulase or endoglucanase activity, have been described in *S. viridis*, and may represent a potential new enzyme source for use in pulp bleaching preparations. In the current study, we reported the cloning, expression, purification, characterization, and pulp biobleaching application of an alkali-tolerant thermostable peroxidase *Svi*DyP from *S. viridis* DSM 43017.

Bioinformatic analysis revealed one unannotated DyP-type peroxidase gene in the genome of *S. viridis* DSM 43017 that had sequence similarity with other DyPs at the primary structure level showing the characteristic conserved residues in the heme-binding site and GXXDG motif [12]. Based on the phylogenetic analysis, *Svi*DyP belongs to subfamily A of DyPs. Compared with other DyPs from subfamily A, the purified recombinant *Svi*DyP, appeared remarkably broad range substrate specificity of which could degrade various dyes, such as triarylmethane dyes, anthraquinonic and azo dyes, at different levels, much better than some enzymes grouped into subfamily A, for example, YcdB [48], TfuDyP [21]. The enzyme could efficiently decolorize triarylmethane dyes to other triarylmethane dye-degrading microorganisms [49], [50], and was more active towards crystal violet [51], [52] and malachite green [51], [53], [54]. *Svi*DyP also showed the ability to degrade anthraquinonic and azo dyes under neutral to alkaline conditions. Hence, such a multifunctional peroxidase with wide substrate specificity was likely capable of decolorizing other, currently unidentified, xenobiotics, Which suggested that *Svi*DyP might be applied directly to solve various environmental and industrial problems, such as the disposal of dye wastewater and pulp biobleaching rather than the DyPs [8], [9] which usually worked under acid condition.

To further explore the feasibility of using *Svi*DyP in biotechnological applications, enzyme stability was evaluated at different pH levels and temperatures. More luckily, *Svi*DyP was very stable over a pH range of 5–10, and retained more than 80% of the maximum residual activity. It also displayed high thermal stability, maintaining 63% of residual activity after 4 h of incubation at 50°C, and more than 60% of residual activity after 2 h of incubation at 60°C. *Svi*DyP showed greater stability than DyP from *Anabaena* sp. [16], which had an activity loss of 90% following 3 h incubation at 50°C. Stability studies showed that *Svi*DyP exhibited a high degree of stability under alkaline pH and high temperatures. This ability to work efficiently under such high pH environments was a characteristic that distinguishes DyPs from most peroxidases [55]. These findings enriched DyP-type peroxidases family's information databases and laid the foundation for the study of structure, function and reaction mechanisms of *Svi*DyP.

Concerning the influence factors of using SviDyP in the further bleaching industrial application, the activation/inactivation effect of the different metal ions and chemical reagents on the enzyme activity was studied. Interestingly, addition of EDTA did not inhibit the enzyme, which suggested that there are no essential ions in the reaction mixture for the enzyme activity [7], [8]. Al^3+^ had similar activating effect between *Svi*DyP and *Pseudomonas aeruginosa* DyPPa [7], probably through promoting the oxidation. SDS resulted in the complete activity loss of *Svi*DyP and DyPPa [7], which demonstrated that SDS could be an important DyP inhibitor. β-mercaptoethanol highly boosted the enzymatic activity presumably by counteracting the oxidation effects of the S–S linkage between cysteine residues [56], [57].

The applicability of *Svi*DyP in biobleaching of kraft pulp, a process of high biotechnological interest, was examined by prebleaching eucalyptus kraft pulp with the purified *Svi*DyP enzyme. Under 65°C, pH 7.0, *Svi*DyP enzyme had positive effects on the pulp bleaching process as well as MnP, while the biobleaching effects of MnP at 25°C, pH 4.5, were better than *Svi*DyP at 65°C, pH 7.0. These data suggest a slightly temperature and pH adjustment were needed in the usage of *Svi*DyP in bleaching pulp. Furthermore, the SEM studies revealed that the existence of local fracture phenomena and the large area of the trench structure containing holes would result in the fiber structure becoming loose, and the internal structure of lignin being fully exposed. This may help the bleaching agent permeate better, and perfects delignification, which will happen later. Thereby, this breakdown in structure means that much less bleaching agent was needed, and less poisoned organochlorine was produced (data not shown).

In addition, the prepared *Svi*DyP peroxidase had hardly some cellulase activity (data not shown), thereby it prevented reduction of fiber strength and viscosity of thick liquid. The enzyme did show significant heat and alkali resistance, and could directly and effectively attack lignin. Therefore, *Svi*DyP is applicable to the process of pre-bleaching paper pulp for industrial production, and shows excellent bleaching effects. With its wide substrate specificities and thermophilic capabilities, *Svi*DyP peroxidase is a promising enzyme with considerable biotechnological and commercial potential, especially in the pulp and paper industry.

## Supporting Information

Figure S1 **Model structure of** ***Svi*** **DyP.** Model structure of SviDyP generated using template sequences from putative uncharacterized protein Sco3963 from *Streptomyces coelicolor* A3 (2) (PDB 4gt2A, GI: 541881521), whose amino acid sequence was 45.56% identical to SviDyP. The α-helices are shown in red and the β-sheets in yellow.
(TIF)

Figure S2 **Paper properties of** ***Svi*** **DyP and chemically-treated eucalyptus kraft pulp.** **a**. paper brightness on various enzyme dosages. **b**. paper kappa number on various enzyme dosages. **c**. paper brightness on various time intervals. **d**. paper kappa number on various time intervals.
(TIF)
