# Supplementary figures and images for: Application of a Novel Alkali-Tolerant Thermostable DyP-Type Peroxidase from Saccharomonospora viridis DSM 43017 in Biobleaching of Eucalyptus Kraft Pulp
Source: PLoS One. 2014 Oct 21;9(10):e110319. doi: 10.1371/journal.pone.0110319 (PMC4204856; doi:10.1371/journal.pone.0110319)

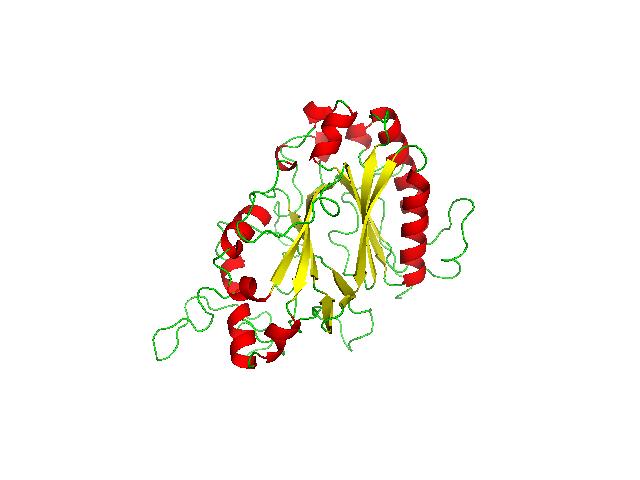

Supplement: Figure S1 — Model structure of Svi DyP. Model structure of SviDyP generated using template sequences from putative uncharacterized protein Sco3963 from Streptomyces coelicolor A3 (2) (PDB 4gt2A, GI: 541881521), whose amino acid sequence was 45.56% identical to SviDyP. The α-helices are shown in red and the β-sheets in yellow. (TIF) [file pone.0110319.s001.tif]

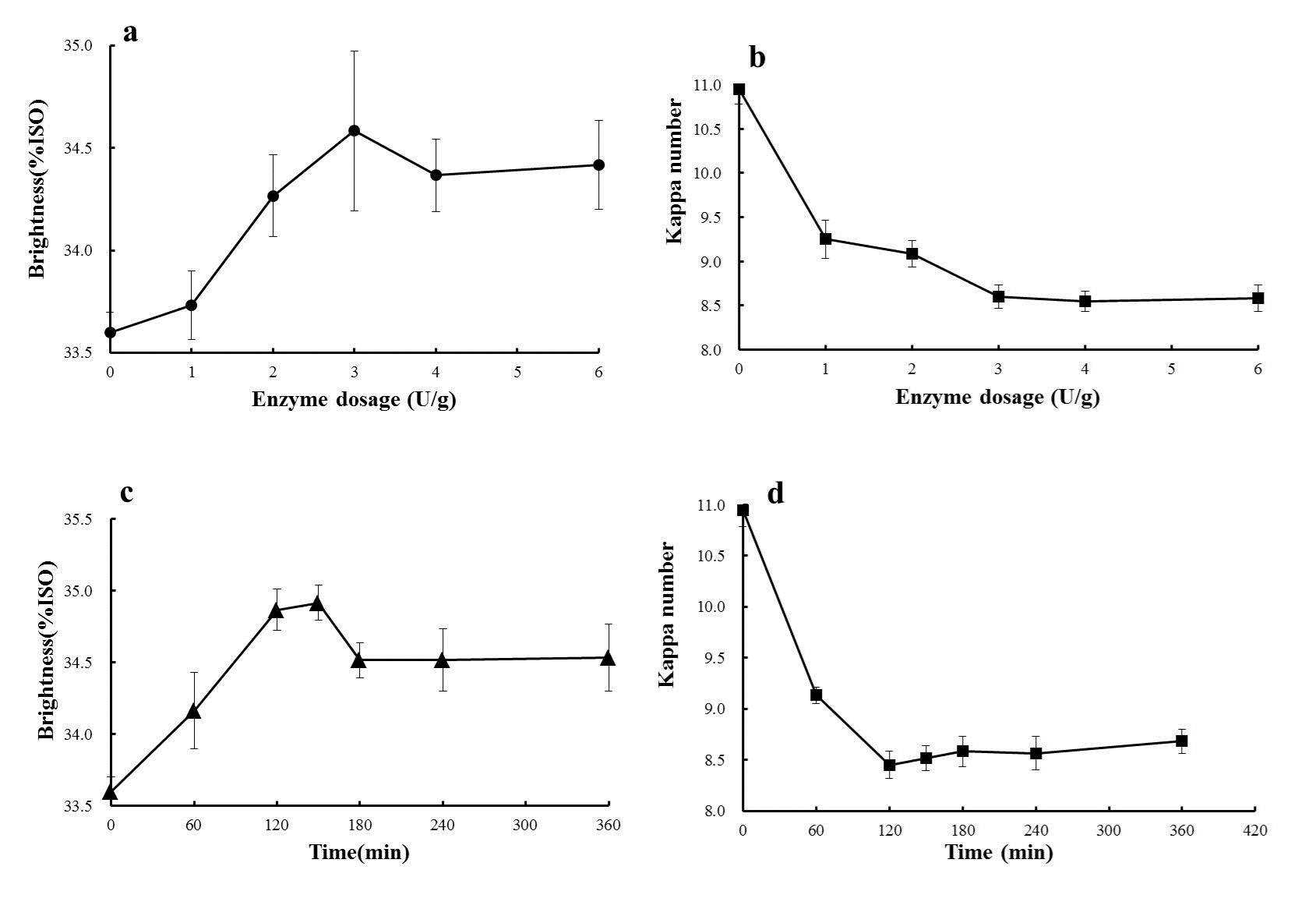

Supplement: Figure S2 — Paper properties of Svi DyP and chemically-treated eucalyptus kraft pulp. a. paper brightness on various enzyme dosages. b. paper kappa number on various enzyme dosages. c. paper brightness on various time intervals. d. paper kappa number on various time intervals. (TIF) [file pone.0110319.s002.tif]
